# Diverging humoral and cellular immune responses due to Omicron—a national study from the Faroe Islands

**DOI:** 10.1128/spectrum.00865-23

**Published:** 2023-11-01

**Authors:** Maria Skaalum Petersen, Laura Pérez-Alós, Sunnvør K. í Kongsstovu, Eina Hansen Eliasen, Cecilie Bo Hansen, Sólrun Larsen, Jóhanna Ljósá Hansen, Rafael Bayarri-Olmos, Jógvan Páll Fjallsbak, Pál Weihe, Peter Garred

**Affiliations:** 1 Department of Research, The National Hospital of the Faroe Islands, Tórshavn, Faroe Islands; 2 Center of Health Science, University of the Faroe Islands, Tórshavn, Faroe Islands; 3 Laboratory of Molecular Medicine, Department of Clinical Immunology, Section 7631, Copenhagen University Hospital, Rigshospitalet, Copenhagen, Denmark; 4 Chief Medical Officer Office, Tórshavn, Faroe Islands; 5 Recombinant Protein and Antibody Unit, Copenhagen University Hospital, Rigshospitalet, Copenhagen, Denmark; 6 Faroese Food and Veterinary Authority, Tórshavn, Faroe Islands; National Taiwan University, Taipei, Taiwan

**Keywords:** humoral immune response, cellular immune response, Omicron, Faroe Islands

## Abstract

Immunity following infection and vaccination with the SARS-CoV-2 Omicron variant is poorly understood. The aim was to investigate immunity assessed with antibody response, neutralizing antibodies (NAbs), and IFN-γ release under different scenarios: in vaccinated and unvaccinated individuals with and without SARS-CoV-2 infection with the Omicron variant. This nationwide single-center study was conducted between January and March 2022, where all convalescent individuals were infected with the Omicron variant and included six study groups: unvaccinated-naïve, unvaccinated convalescent, vaccinated-naïve (second dose), vaccinated-naïve (third dose), vaccinated convalescent (second dose), and vaccinated convalescent (third dose). Antibody responses were assessed by determining receptor binding domain-specific antibodies and NAbs levels in serum, and IgG in saliva. T-cell responses in whole blood were measured as IFN-γ levels released after stimulation with spike peptides. We found that the humoral response against the spike protein was higher among vaccinated-naïve than unvaccinated convalescent. Unvaccinated with and without infection had comparable low humoral responses, while those vaccinated with a second or third dose, independent of infection status, had increasingly higher levels. Only 22% of the unvaccinated convalescent individuals mounted consistent detectable humoral responses following Omicron infection. However, 98% had spike peptide T-cell responses assessed by IFN-γ release. In conclusion, primary Omicron infection mounts a low humoral immune response, significantly enhanced by prior vaccination. Omicron infection induced a robust T-cell response in both unvaccinated and vaccinated, demonstrating that the evasive immune potential of primary Omicron infection affects humoral immunity more significantly than T-cell immunity.

**IMPORTANCE:**

The immunity following infection and vaccination with the SARS-CoV-2 Omicron variant is poorly understood. We investigated immunity assessed with antibody and T-cell responses under different scenarios in vaccinated and unvaccinated individuals with and without Omicron infection. We found that the humoral response was higher among vaccinated-naïve than unvaccinated convalescent. Unvaccinated with and without infection had comparable low humoral responses, whereas vaccinated with a second or third dose, independent of infection status, had increasingly higher levels. Only a minor fraction of unvaccinated individuals had detectable humoral responses following Omicron infection, while almost all had positive T-cell responses. In conclusion, primary Omicron infection mounts a low humoral immune response, enhanced by prior vaccination. Omicron infection induced a robust T-cell response in both unvaccinated and vaccinated, demonstrating that immune evasion of primary Omicron infection affects humoral immunity more than T-cell immunity.

## INTRODUCTION

The continuing evolution of severe acute respiratory syndrome coronavirus 2 (SARS-CoV-2) has given rise to several novel variants (https://covid19.who.int/) characterized by sets of mutations, raising concerns about possible immune evasion and increased transmissibility (1). Omicron BA.1 lineage of SARS-CoV-2 emerged in late 2021 and quickly became dominant, in part because of a large number of mutations that allowed escape from existing antibodies. The Omicron variant includes different sub-lineages that have been shown to transmit more readily due to the extensive mutations found in its spike protein which raised concerns that the efficacy of current COVID-19 vaccines and antibody therapies might be compromised (2, 3).

Progress has been made in understanding immune responses to SARS-CoV-2 infection and COVID-19 vaccination. Robust and broad immune responses precede individuals’ recovery (4). While antibodies produced by B-cells, especially neutralizing antibodies (NAbs), generate immunity and prevent SARS-CoV-2 infection by blocking infection and clearing pathogens, T-cells appear to limit disease severity, reduce its duration, and drive rapid recovery (4, 5). Many studies, including ours (6), have reported long-lasting but decreasing circulating antibodies over time in convalescent individuals. Still, recent studies point to a robust and durable T-cell immunity, suggesting that this may be a more reliable marker of prior infection than the humoral response (7 – 9). Therefore, measuring antibody production and T-cell responses may be necessary to better characterize the immunity against SARS-CoV-2.

There is increasing evidence that individuals who previously recovered from COVID-19 have enhanced immune responses after vaccination (hybrid immunity) compared to naïve-vaccinated individuals (10, 11). However, Omicron seems less sensitive to NAb responses induced by vaccination and prior infection than previous variants (3, 12 – 14). It is, however, not entirely clear how different combinations of infection with Omicron and/or infection shape the immune response. The aim of this nationwide single-center study was to investigate the influence on humoral receptor binding domain (RBD)-specific antibodies and cellular T-cell anti-spike immunity in vaccinated and unvaccinated individuals with and without Omicron SARS-CoV-2 infection.

## RESULTS

A total of 493 individuals participated in this study and were divided into six groups based on SARS‐CoV‐2 infection and/or vaccination: (i) unvaccinated SARS-CoV-2-naïve individuals (*n* = 88); (ii) unvaccinated SARS-CoV-2 convalescent individuals (*n* = 82); (iii) vaccinated SARS-CoV-2-naïve individuals (second dose; *n* = 11); (iv) vaccinated SARS-CoV-2-naïve individuals (third dose; *n* = 103); (v) vaccinated convalescent individuals (second dose; *n* = 102); and (vi) vaccinated convalescent individuals (third dose; *n* = 107). Table 1 depicts the characteristics of the six study groups.

**TABLE 1:** Characteristics of the study groups, sampled from January to March 2022

	Group 1 Unvaccinated naïve		Group 2 Unvaccinated convalescent^*b*^		Group 3 Vaccinated naïve (second dose)		Group 4 Vaccinated naïve (third dose)		Group 5 Vaccinated convalescent^*b*^ (second dose)		Group 6 Vaccinated convalescent^*b*^ (third dose)		*P*-value^*c*^
Total (*n*)	*n = 88*		*n = 82*		*n = 11*		*n = 103*		*n = 102*		*n = 107*		
Sex, *n* (%)													0.02
Female	44	50	56	68.3	8	72.7	66	64.1	75	73.5	69	64.5	
Male	44	50	26	31.7	3	27.3	37	35.9	27	26.5	38	35.5	
Age (years), median (5%–95% percentile)	41.4	15.6–70.9	42.4	19.1–69.1	45.8	20.3–70.0	53.2	28.2–70.3	36.3	17.5–60.3	50.1	28.6–73.1	<0.001
BMI (kg/m^2^), median (5%–95% percentile)	*n = 78*		*n = 76*		*n = 10*		*n = 99*		*n = 102*		*n = 107*		0.05
	25.9	17.8–37.3	25.7	19.8–37.1	23.4	18.6–34.8	27.1	21.5–35.6	25.7	19.8–35.7	26.9	21.0–34.7	
Smoking, *n* (%)	*n = 86*		*n = 81*		*n = 10*		*n = 85*		*n = 102*		*n = 107*		0.02
Ever	49	57.0	39	48.1	8	80.0	63	61.8	45	44.1	59	55.1	
Never	37	43.0	42	51.9	2	20.0	39	38.2	57	55.9	48	44.9	
Daily medication use, *n* (%)	*n = 79*		*n = 76*		*n = 11*		*n = 100*		*n = 100*		*n = 106*		0.02
Yes	24	30.4	15	19.7	3	27.3	45	45.0	29	29.0	39	36.8	
No	55	69.6	61	80.3	8	72.7	55	55.0	71	71.0	67	63.2	
Self-reported chronic disease^*a*^, *n* (%)	*n = 74*		*n = 65*		*n = 10*		*n = 85*		*n = 92*		*n = 105*		0.005
Yes	27	36.5	24	36.9	3	30.0	50	58.8	35	38.0	56	53.3	
No	47	63.5	41	63.1	7	70.0	35	41.2	57	62.0	49	46.7	
Days from RT-PCR test/omicron infection to blood sample, median (5%–95% percentile)^*d*^			28	8–56					37	12–60	30	8–58	*<0.001*
Days between last dose and blood sample, median (5%–95% percentile)					242	77–347	67	20–118	226	102–283	79	38–123	*0.004*
Days between last dose to RT-PCR-test/omicron infection, median (5%–95% percentile)									183	62–244	47	6–106	<0.001

^*a*^ Asthma, heart disease, carnitine transporter deficiency, inflammatory bowel disease, hypertension, hypercholesterolemia, chronic obstructive pulmonary disease, and type 2 diabetes.

^*b*^ All convalescent individuals were infected between 1 Jan 2022 and 7 March 2022.

^*c*^ Categorical χ^2^ ; continuous: non-parametric, Kruskal-Wallis H.

^*d*^ Missing for three individuals in group 2, one individual in group 4, and one individual in group 5.

For each sample, we quantitatively measured Wildtype-RBD-specific IgG, IgA, and IgM levels using an enzyme-linked immunosorbent assay (ELISA)-based assay in serum and IgG in saliva with a Luminex-based assay, virus-NAbs with an ELISA-based pseudo-neutralizing, and IFN-γ release after stimulating T-cells against Wildtype spike peptides using S-ELISA. Finally, in a subgroup, we measured serum IgG levels and NAb capacity using BA.1 and BA.2 RBD for correlation with Wildtype-RBD.

**Fig 1:**
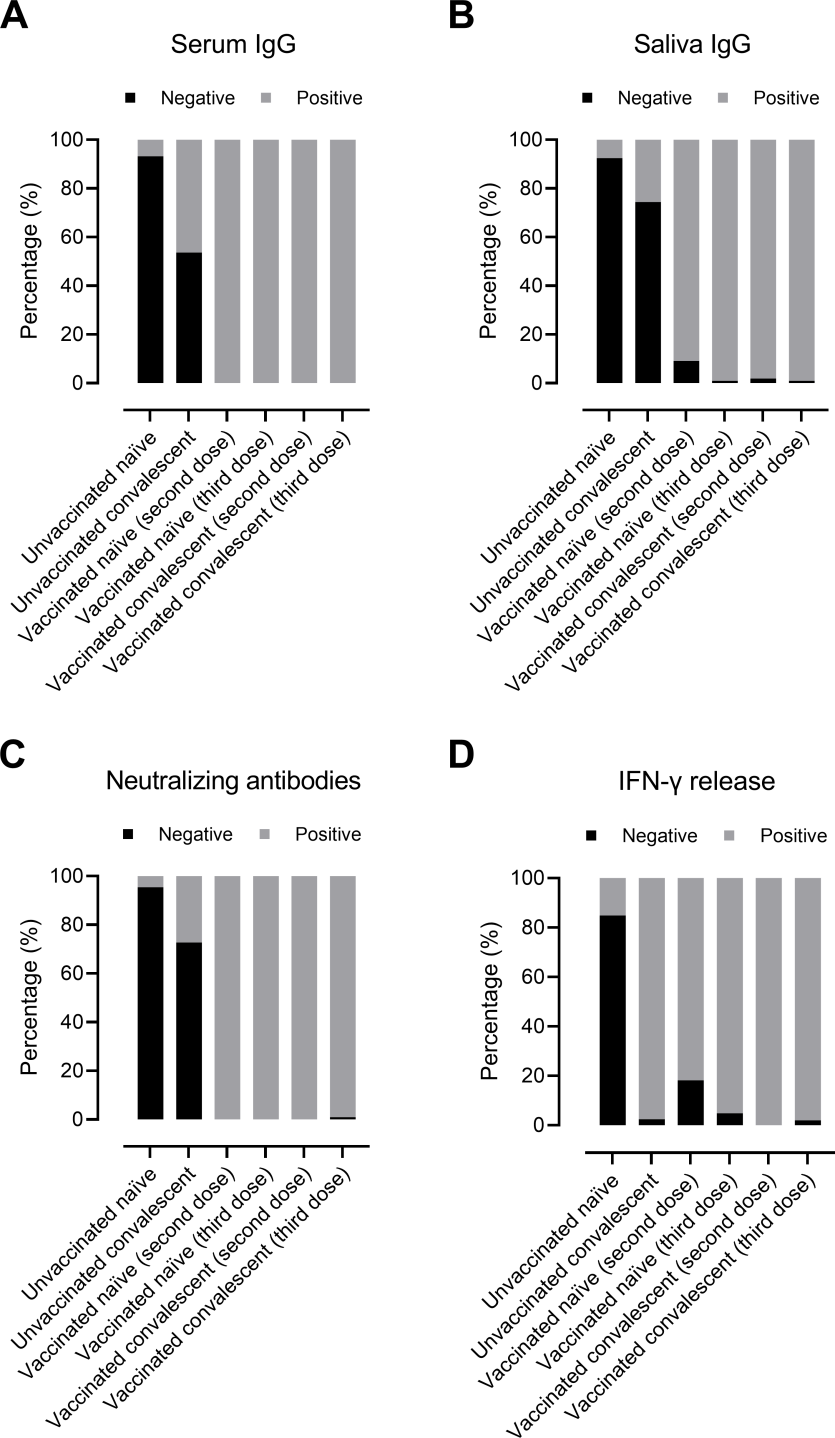
Detected humoral antibody and cellular responses in study groups (represented as percentage) divided into negative or positive responses. Serum RBD-IgG antibodies (**A**), saliva RBD-IgG antibodies (**B**), NAbs (**C**), and IFN-γ release after stimulating T-cells against spike protein (**D**). The participants are divided into six groups based on SARS‐CoV‐2 infection and/or vaccination: (i) unvaccinated SARS-CoV-2-naïve individuals (*n* = 88); (ii) unvaccinated SARS-CoV-2 convalescent individuals (*n* = 82); (iii) vaccinated SARS-CoV-2-naïve individuals (second dose; *n* = 11); (iv) vaccinated SARS-CoV-2-naïve individuals (third dose; *n* = 103); (v) vaccinated convalescent individuals (second dose; *n* = 102); and (vi) vaccinated convalescent individuals (third dose; *n* = 107). Experimental analyses were performed using Wildtype-RBD.

Regardless of SARS‐CoV‐2 infection status, almost all vaccinated individuals had detectable serum and saliva IgG antibodies, NAbs and IFN-γ released from T-cells (Fig. 1). As expected, the majority of unvaccinated SARS-CoV-2-naïve individuals (group 1) did not have a detectable immune response (Fig. 1). In this group, some individuals who were thought not to have been infected with SARS-CoV-2 had detectable IgG levels indicating that they probably had been infected previously, most likely asymptomatic. Among vaccinated naïve individuals (third dose; group 4), all individuals had detectable IgG and NAbs, while IFN-γ release was below the threshold for seven individuals. However, only 46% of unvaccinated convalescent individuals (group 2) had detectable IgG antibodies, and only 27% had NAbs, whereas 98% had detectable IFN-γ levels.

**Fig 2:**
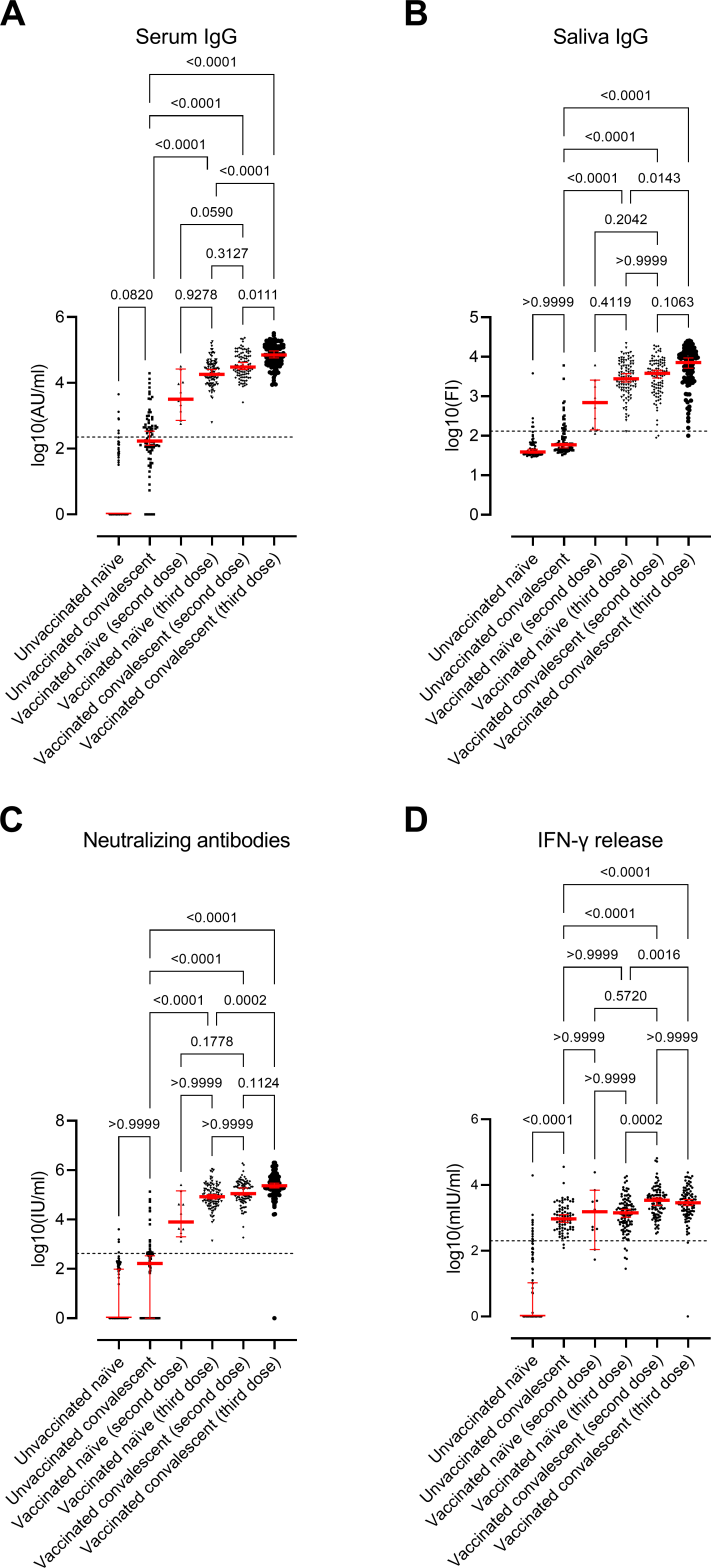
Immune response in study groups. Scatter plots display the median values and 95% CI (red line and bars): Serum RBD-IgG antibodies (**A**), saliva RBD-IgG antibodies (**B**), NAbs (**C**), and IFN-γ release after stimulating - cells against spike protein (**D**). The participants are divided into six groups based on SARS‐CoV‐2 infection and/or vaccination: (i) unvaccinated SARS-CoV-2-naïve individuals (*n* = 88); (ii) unvaccinated SARS-CoV-2 convalescent individuals (*n* = 82); (iii) vaccinated SARS-CoV-2-naïve individuals (second dose; *n* = 11); (iv) vaccinated SARS-CoV-2-naïve individuals (third dose; *n* = 103); (v) vaccinated convalescent individuals (second dose; *n* = 102); and (vi) vaccinated convalescent individuals (third dose; *n* = 107). The horizontal line represents the threshold for assay positivity (225 AU/mL for IgG, 132 FI for saliva IgG, 420 IU/mL for NAbs, and 200 mIU/mL for IFN-γ). *P* < 0.05 was considered statistically significant. Experimental analyses were performed using Wildtype-RBD.

All groups had a significant overall difference regarding the serum and saliva IgG antibody levels, NAbs, and IFN-γ levels (Kruskal–Wallis test, *P* < 0.0001; Fig. 2). Levels of antibodies and IFN-γ release in each group are presented in Table S1.

Serum IgG antibodies differed significantly in all groups (Kruskal-Wallis test, *P* < 0.0001, Fig. 2A), where the highest values were observed in the vaccinated convalescent individuals (third dose) group (group 6) and the lowest in the unvaccinated naïve individual’s group (group 1; Fig. 2A). Unvaccinated convalescent individuals (group 2) had significantly higher IFN-γ levels than unvaccinated naïve individuals (group 1; *P* < 0.0001) but not regarding serum IgG, saliva IgG, and NAbs levels. On the other hand, unvaccinated convalescent individuals (group 2) had significantly lower serum IgG, saliva IgG, and NAbs levels than vaccinated naïve individuals (third dose; group 4; *P* < 0.0001 for all) and vaccinated convalescent individuals (both two and third dose; groups 5 and 6; *P* < 0.0001 for all). IFN-γ levels were significantly higher in groups 5 and 6 compared to group 2 (*P* < 0.0001). However, similar IFN-γ levels were observed for unvaccinated convalescent individuals (group 2) and vaccinated naïve individuals (second and third dose; groups 3 and 4), suggesting a similar cellular response in unvaccinated individuals infected with Omicron and vaccinated naïve individuals.

The correlation between IgG and NAbs was high in the vaccinated groups (groups 3–6) (Spearman’s correlation, rho > 0.76), regardless of infection status, while it was lower in unvaccinated convalescent individuals (group 2; rho = 0.38; Fig. S1). Yet, the correlation between saliva and serum IgG was similar in all groups (rho ~0.50) (Fig. S1). However, there was no correlation between IFN-γ levels and IgG and NAbs in the convalescent groups (groups 2, 5, and 6; rho <0.26) but only in the group with vaccinated SARS-CoV-2-naïve individuals (third dose; group 4; rho >0.43; Fig. S1).

Among the unvaccinated SARS-CoV-2 convalescent individuals (group 2), all but two individuals had detectable IFN-γ levels (97%), while only 46% had detectable serum IgG levels. Of those with detectable IgG, only 14 also had NAbs, while four others had both detectable NAbs and IFN-γ release but not IgG. Overall, consistency in humoral immune response was only 22% in this group. This is also evident looking at correlations where much less correlation is observed in this group compared to the other groups, e.g., the correlation between IgG and NAbs was rho ~0.8 in groups with vaccinated individuals (groups 4, 5, and 6) and only rho ~0.38 among unvaccinated SARS-CoV-2 convalescent individuals (group 2; Fig. S1).

**Fig 3:**
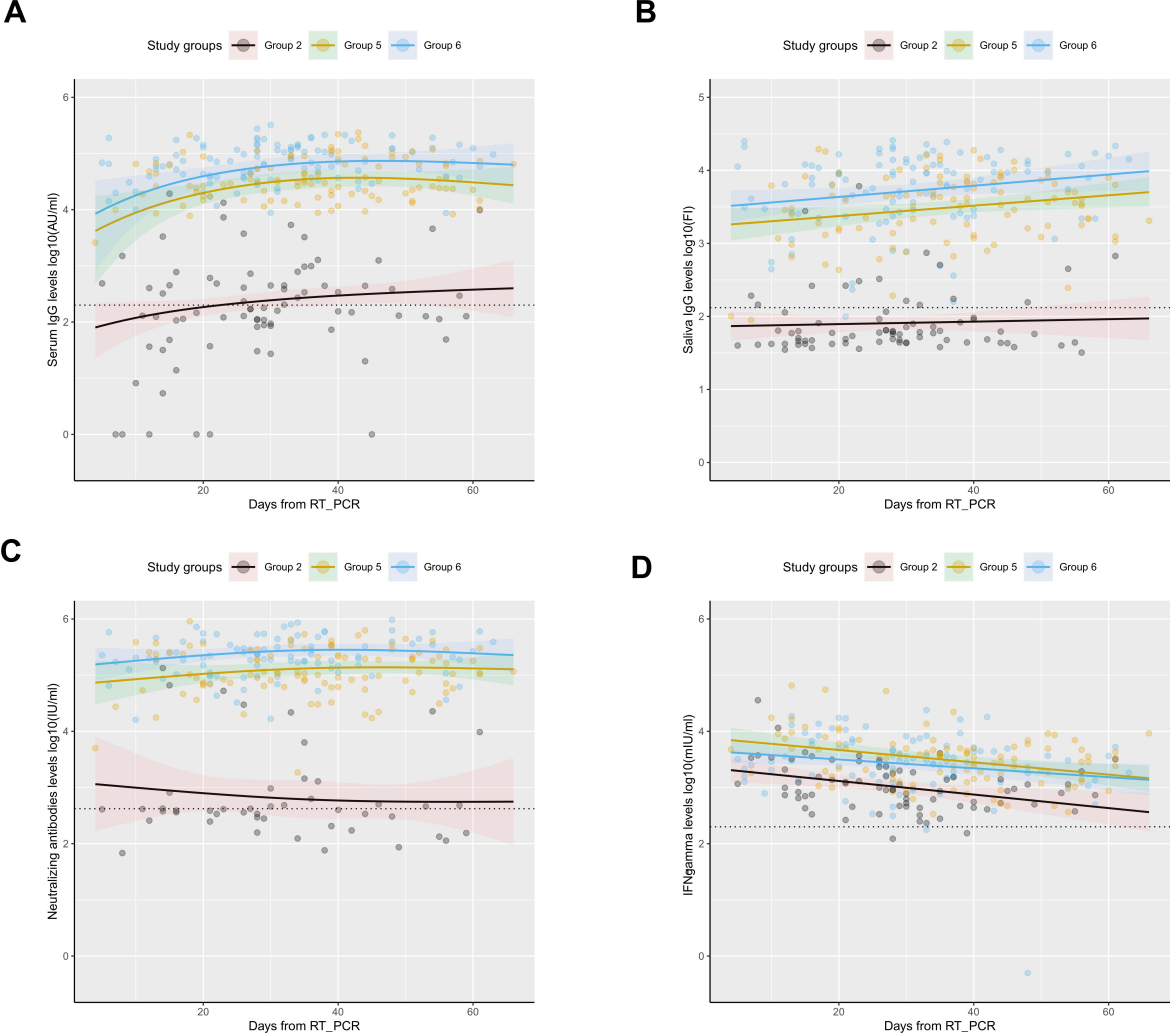
Antibody levels and IFN-γ levels dynamics over time in convalescent individuals using mixed models. Distribution of (**A**) serum IgG levels, represented in log10(AU/mL), (**B**) saliva IgG levels, represented in log10(FI), (**C**) NAbs levels, represented in log10(IU/mL); and (**D**) IFN-γ levels, represented in log10(mIU/mL) over time (days from positive RT-PCR result). Circles represent observed levels for (**A**) serum IgG, (**B**) saliva IgG, (**C**) NAbs, and (**D**) IFN-γ. Solid lines represent predicted levels for (**A**) serum IgG levels, (**B**) saliva IgG levels, (**C**) NAbs levels, and (**D**) IFN-γ levels. Black, yellow, and blue colors represent unvaccinated convalescent individuals (group 2), vaccinated convalescent individuals (second dose; group 5), and vaccinated convalescent individuals (third dose; group 6), respectively. The horizontal dotted line represents the assay positivity threshold. The confidence interval (95%) is represented by the shadowed areas. The center for the confidence interval is the predicted (mean) values.

Samples were collected over a period of 2 months for the majority of participants, also in the group of unvaccinated SARS-CoV-2 convalescent individuals (group 2). To examine whether the lack of positive humoral responses in the group of unvaccinated convalescent individuals (group 2) resulted from a too-early sample collection following infection onset, we modeled the humoral and cellular responses in convalescent individuals (groups 2, 5, and 6) to evaluate if the dynamics changed over time. Fig. 3 shows the serum IgG, saliva IgG, NAbs, and IFN-γ levels over time since the day of RT-PCR positive result (Fig. 3A, B, C and D, respectively), where, as mentioned above, the vaccinated individuals presented significantly higher levels (*P* < 0.0001 for all antibodies and IFN-γ levels). However, no significant differences in dynamics over time were observed between the groups in relation to serum IgG, saliva IgG, NAbs, and IFN-γ indicating that the trends for the convalescent groups are similar regardless of vaccination status. Nevertheless, the IFN-γ decreases over time for all three convalescent groups. Zero observations for NAbs in group 2 were excluded to allow model fit. Thus, the waning of antibody responses does not seem to be a likely explanation to the observed lack of IgG response in the unvaccinated convalescent individuals.

**Fig 4:**
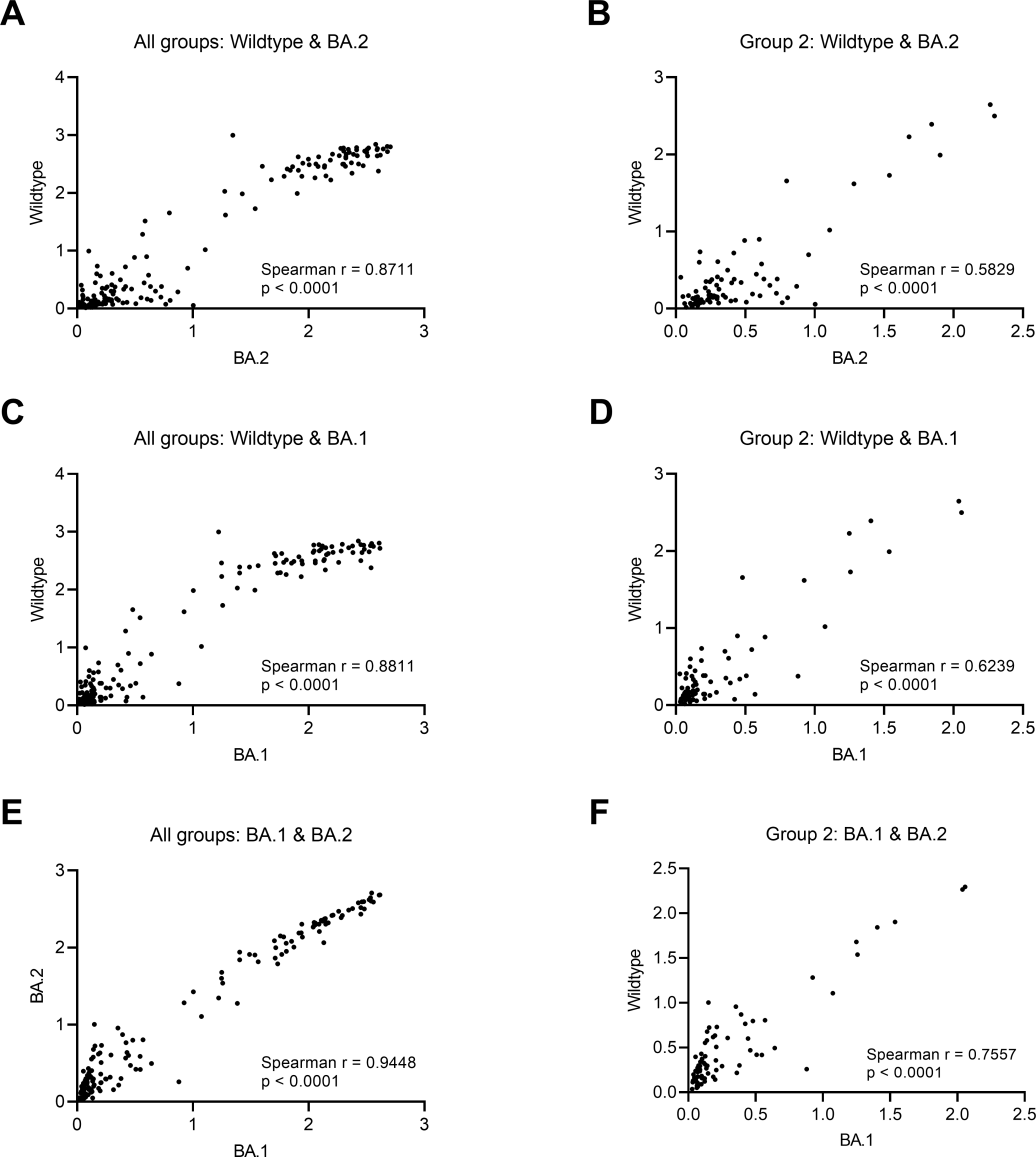
Correlation between Wildtype RBD and BA.1 and BA.2 RBD. (**A, C, and E**) Spearman correlation between Wildtype RBD and BA.2 and BA.1 RBD for all samples in all study groups. (**B, D, and F**) Spearman correlation between Wildtype RBD and BA.2 and BA.1 RBD for all samples for only group 2 (unvaccinated convalescent individuals). *P* < 0.05 was considered statistically significant. Optical densities are compared.

To further elucidate a potential explanation for the high proportion of non-detectable antibodies among unvaccinated SARS-CoV-2 convalescent individuals (group 2), we explored a slightly altered assay using BA.1 and BA.2 RBD to assess if with the current method used, Wildtype-RBD, the antibody levels were underestimated after Omicron infection. Thus, we ran 162 samples using BA.1 and BA.2 RBD, including the group 2 samples (unvaccinated SARS-CoV-2 convalescent) and randomly selected samples from the other groups. We observed a high correlation between the three RBD tested (Fig. 4A through F). Based on these results, we can conclude that we do not gain further information by including the variant assays, as most individuals have similar antibody results in all assays. In addition, in a subset of samples matched on age, sex, and time after infection or vaccination, we studied the neutralization capacity against Wildtype, BA.1, and BA.2 RBD (Fig. S2). We found no significant difference between the three RBD variants in the unvaccinated Omicron-convalescent group (Friedman test, Fig. S3A). However, the neutralization capacity was significantly higher when using Wildtype-RBD compared to both BA.1 and BA.2 RBD in the unvaccinated Wildtype-convalescent group (Fig. S2). Moreover, we found a significant correlation between IgG levels and neutralization capacity among the three RBD variants tested (rho >0.55, Fig. S3B through D).

Additionally, since the sample collection was performed shortly after infection onset, we have compared IgG dynamics after Omicron infection with Wildtype infection over time using generalized linear mixed models to further elucidate whether an increase in IgG levels after Omicron infection could occur (Fig. S4). Wildtype-infected individuals have been previously described (6), and demographic characteristics can be found in Table S2. Based on the IgG dynamics after Wildtype infection and the significantly lower antibody IgG response mounted after Omicron infection (*P* < 0.0001), it is rather unlikely that an increase in IgG levels occurred after 60 days from Omicron infection onset.

**Fig 5:**
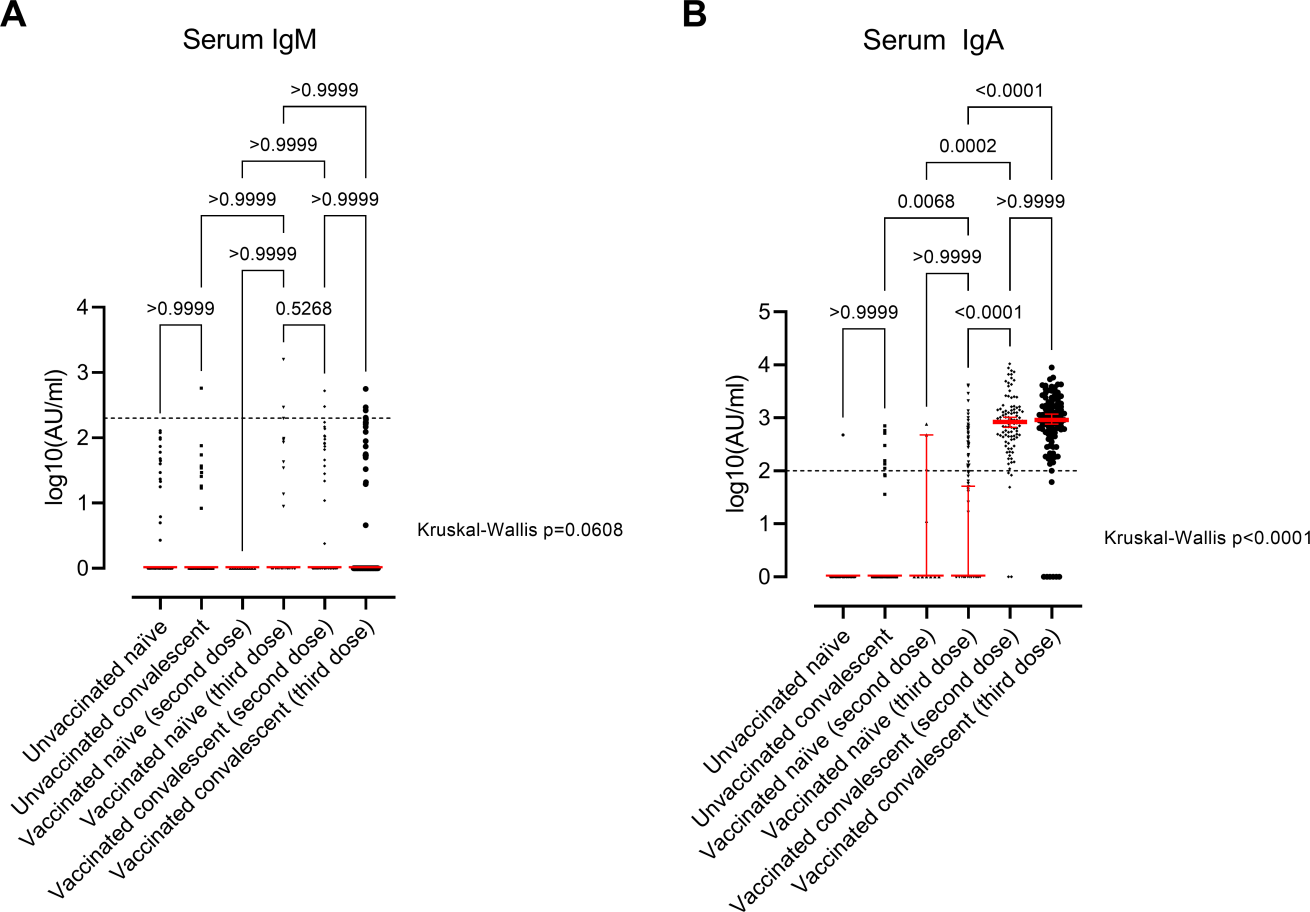
Immune response in study groups. Scatter plots display the median values and 95% confidence interval (CI; red line and bar) of serum IgM antibodies (**A**) and serum IgA antibodies (**B**). The participants are divided into six groups based on SARS‐CoV‐2 infection and/or vaccination: (i) unvaccinated naïve individuals (*n* = 88); (ii) unvaccinated convalescent individuals (*n* = 87); (iii) vaccinated naïve individuals (third dose; *n* = 11); (iv) vaccinated naïve individuals (third dose; *n* = 103); (v) vaccinated convalescent individuals (second dose; *n* = 104); and (vi) vaccinated convalescent individuals (third dose; *n* = 107). *P* < 0.05 was considered statistically significant by Kruskal–Wallis test followed by multiple comparisons with Dunn’s correction. The horizontal line represents the threshold for assay positivity (200 AU/mL for IgM and 100 AU/mL for IgA).

Regarding the other antibody isotypes quantified, we observed a significant difference between the study groups in serum IgA levels (Kruskal-Wallis test, *P* < 0.0001) but not serum IgM levels (Kruskal-Wallis test, *P* = 0.06; Fig. 5). Of particular interest, only individuals vaccinated and infected (hybrid immunity) mounted a significant IgA response (Kruskal-Wallis test, *P* < 0.0001, Fig. 5).

## DISCUSSION

In this study of the immune response following infection with the SARS-CoV-2 Omicron variant and vaccination in the Faroe Islands, serum and saliva IgG antibody levels, NAbs, and T-cell activity showed a significant difference between all groups studied.

We found that only half of the unvaccinated SARS-CoV-2 convalescent individuals mounted detectable but low IgG levels compared to both vaccinated naïve and vaccinated convalescent individuals. Less than 23% of these individuals had measurable NAbs and saliva antibodies, whereas the vast majority had T-cell activity assessed by IFN-γ release. The Omicron variant exhibits over 30 mutations in the spike protein, including at least 15 mutations in the RBD (11). We speculated if the lack of antibodies in a high proportion of unvaccinated convalescent individuals was due to the antibody assays based on the original Wuhan-Hu-1 strain (Wildtype) and, therefore, might not accurately capture infections with Omicron variant. However, our exploratory analyses with BA.1 and BA.2 RBD, i.e., adjusted to detect antibodies against Omicron, showed similar results, both for serum IgG and NAb capacity. This indicates that the method used in this study (Wildtype RBD) is comparable to measure Omicron immune responses in this cohort and thus cannot explain the observed low frequency of IgG seropositivity. However, some studies do report reduced sensitivity of commercial antibodies assays contrary to our results (15, 16). The lack of NAbs and saliva IgG levels in the same individuals further supports the observation. Another potential explanation that we explored was if the samples were taken too soon after infection, and the antibody production had not been initiated. However, our time analyses, including only individuals infected in 2022, showed no significant differences in serum/saliva IgG levels, NAbs levels, and IFN-γ levels investigated from the time of positive RT-PCR result to the time of sampling both in unvaccinated convalescent individuals and the vaccinated convalescent individuals. Moreover, similar IgG dynamics trends were observed between Omicron- and Wildtype-infected unvaccinated individuals, and others reported a high IgG seroconversion but weak in unvaccinated Omicron-infected individuals (17). Thus, our results indicate that the Omicron variant does not mount a robust humoral response in unvaccinated individuals, but robust cellular responses are indeed mounted. Other studies report similar findings. One study reported 73.3% of samples exhibiting no measurable neutralizing activity for Omicron, which agrees with our study (18). Another study (19) found that Omicron infection in non-vaccinated individuals did not result in any significant NAbs production within a time window of 2 weeks, suggesting that the initial exposure to Omicron spike proteins does not elicit a substantial immune response. Additionally, despite discrepancies described between different studies (20), we found a correlation between IgG levels and NAbs in unvaccinated convalescent individuals. This underscores the necessity to further investigate Omicron-specific immune responses to evaluate correlates of protection in the Omicron era.

We find that Omicron infection in vaccinated individuals elicits NAbs titers as previously described (21, 22). Higher NAbs titers were observed among those with hybrid immunity (i.e., immunity conferred by the combination of infection and vaccination), especially those with a third dose of vaccine. This is in line with a Korean study where Omicron infection following three-dose vaccination induced robust NAbs that were broadly reactive and more potently protective (23). Their findings also suggest that these antibodies may neutralize both former and novel SARS-CoV-2 variants (23). The level and strength of SARS-CoV-2 NAbs in naïve vaccinated individuals were lower than in convalescent vaccinated individuals, which others also have reported (24). IgG levels were significantly different in all groups, with similar higher levels among those with hybrid immunity, more pronounced after third dose vaccinations in line with a recent finding by Karachaliou et al. (25).

We found that NAbs against Omicron are weakest in unvaccinated convalescent and vaccinated SARS-CoV-2-naïve individuals compared to hybrid immune individuals, as also observed and reported by Carreño et al. (18). IgG antibody responses and NAbs were higher among vaccinated SARS-CoV-2-naïve than unvaccinated convalescent individuals. This is in line with a study that concludes that vaccination is superior to prior infection in eliciting innate and humoral immune responses in Omicron-infected patients. Unvaccinated individuals with and without previous infection had comparable low NAbs levels, while vaccinated individuals, independent of infection status, all produced robust responses even though they were significantly different. By contrast, this was not observed for IFN-γ release, where the levels among unvaccinated convalescent and vaccinated SARS-CoV-2-naïve individuals were similar.

The T-cell activity was assessed as IFN-γ release, which has been shown to be a reliable method of quantifying the T-cell response after SARS-CoV-2 infection or vaccination. However, a study from Spain showed that in convalescent patients, the sensitivity is largely dependent on disease severity and time since primary infection (26). We observed a decline in IFN-γ levels depending on the days between infection and blood sampling. Nevertheless, we observed that almost all vaccinated infection-naïve had a robust response. Thus, we assume that the time frame in our study was not a problem.

IgA is the most abundant immunoglobulin isotype on mucosal surfaces. Thus, experimental work has shown that to achieve protection against transmission of SARS-CoV-2, locally applied vaccines in the airways might be superior to current intramuscular vaccines (27). Consistent with this notion, we have shown that the serum IgA response was substantially increased in vaccinated healthcare employees infected with previous SARS-CoV-2 strains (28, 29). Here, we show that only hybrid immune individuals mount a positive systemic IgA response. This is surprising since we had expected to observe IgA responses after natural Omicron infection. Nevertheless, despite the lack of detectable IgA antibodies after natural Omicron infection, significant priming is necessary to induce a systemic IgA response after vaccination. Based on this observation, it might be suggested that a combination strategy including mucosal and centrally administrated vaccines could be optimal for sterilizing protection against SARS-CoV-2.

One strength of this study is the inclusion of non-vaccinated individuals. The vast majority of cases have RT-PCR-confirmed SARS-CoV-2 caused by the Omicron variant, and all have received the same vaccination scheme (Pfizer/BioNTech, BNT162b2). This assumption is reasonable as partial sequencing of a representative subset of samples during January 2022 showed that almost all cases in the Faroe Islands were infected with the BA.1 or BA.2 variants of Omicron (30). Another strength is the use of standardized antibody and IFN-γ releases serological platforms to accurately measure antibody and T-cell responses in diverse groups assessing immunity under different scenarios in vaccinated and unvaccinated, infection-naïve, and convalescent individuals. However, it is pertinent to mention several limitations of the study. We did not have access to disease severity information. As a cross-sectional study design, this did not allow us to evaluate long-term immunological memory after SARS-CoV-2 Omicron infection or vaccination. Therefore, as unvaccinated Omicron-infected samples were collected up to 60 days from infection onset, we cannot accurately determine whether these individuals develop a higher immune response after this date. It is important to remark on the limited power in the IFN-γ, serum IgM, and serum IgA results in group 3 due to the sample size. The high acceptance of the vaccine booster combined with the high viral transmission of the Omicron variant made it difficult to identify and recruit to the study uninfected individuals who received only two vaccine doses. In addition, the methods we used measured antibody levels against Wildtype-RBD, which could underestimate immune responses mounted after Omicron infection. However, we assessed the difference in assay performance using BA.1 and BA.2 RBD instead of Wildtype-RBD in a subset of samples and observed a high correlation between the three RBD variants tested.

In conclusion, primary Omicron infection mounted only a negligible humoral immune response but was significantly enhanced by prior vaccination. By contrast, primary Omicron infection induced a robust T-cell response in both unvaccinated and vaccinated, demonstrating that the evasive immune potential of Omicron affects humoral immunity more than cellular immunity. Only hybrid immunity mounted a significant systemic IgA response, suggesting that local mucosal immune priming might be important to consider for further vaccine development.

## MATERIALS AND METHODS

### Study groups

In this cross-sectional, nationwide study from the Faroe Islands, participants were recruited from 26 January to 15 March 2022 into six different groups: (i) unvaccinated SARS-CoV-2-naïve individuals; (ii) unvaccinated SARS-CoV-2 convalescent individuals; (iii) vaccinated SARS-CoV-2-naïve individuals (second dose); (iv) vaccinated SARS-CoV-2-naïve individuals (third dose); (v) vaccinated SARS-CoV-2 convalescent individuals (second dose); and (vi) vaccinated SARS-CoV-2 convalescent individuals (third dose). The participants were infected and confirmed with RT-PCR, between 1 January and 7 March 2022; partial sequencing of representative samples in January 2022 in the Faroe Islands showed that more than 95% of all SARS-CoV-2 cases in the Faroe Islands were of the BA.1 and BA.2 Omicron variant, indicating that convalescent individuals included in this study have been infected primarily with the Omicron variant (30). Pfizer-BioNTech vaccine (BNT162b2) was the only vaccine administered in the Faroe Islands, and thus, all vaccinated participants received this vaccine. Individuals receiving less than two vaccination doses were excluded (*n* = 1).

Recruitment was performed in different ways. Participants with SARS-CoV-2 RT-PCR-confirmed infection in January 2022 or onward were mainly recruited through the Chief Medical Officer’s office, which was in contact with all infected cases in the Faroe Islands (31). The Chief Medical Officer’s office sent an email to individuals infected in January and February 2022 on our behalf with an invitation to participate in this study and asking them to contact us if they wanted to participate. Recruiting vaccinated individuals with a second or third dose and unvaccinated SARS-CoV-2-naïve subjects was more challenging. To reach these individuals, we used social media and participated in local radio to inform about the project and ask eligible people to contact the research team if they volunteered to participate.

Participants in the study delivered a blood sample and a saliva sample for assessment of humoral antibody and cellular T-cell anti-spike immunity. They answered a short background questionnaire providing information about SARS-CoV-2 infection, vaccination status, education, employment, smoking habits, height, weight, and selected chronic diseases (asthma, heart disease, carnitine transporter deficiency, inflammatory bowel disease, hypertension, hypercholesterolemia, chronic obstructive pulmonary disease, and type 2 diabetes mellitus), medication use, and self-assessed health.

The Faroese Ethics Committee approved the study; all participants voluntarily participated and provided written informed consent.

### Determination of antibody levels in serum

Quantitative determination of circulating IgG, IgM, and IgA against SARS-CoV-2 Wildtype spike (S) protein RBD (Wildtype-RBD) was performed using an in-house ELISA-based assay as described previously (32). The threshold for assay positivity was defined as 225 arbitrary units (AU)/mL, 200 AU/mL, and 100 AU/mL for IgG, IgM, and IgA, respectively.

Additionally, we determined circulating IgG levels as described above using BA.1 and BA.2 RBD (both from Acrobiosystems, USA) instead of Wildtype-RBD in a subset of samples (81 individuals from group 2 and 19–21 individuals from each of the other groups) to assess the difference in assay performance, if any, due to solely infection with the Omicron variant.

### Determination of antibody levels in saliva

Saliva samples were collected using Oracol-tubes (Malvern Medical Developments, Great Britain) according to the manufacturer’s instructions, followed by centrifugation at 1500 G for 10 min. The supernatant was collected and stored at −20°C until its analysis. IgG quantification was performed using the Luminex 200 platform to be described elsewhere (33). In brief, Wildtype-RBD-coupled beads in Bio-Plex plates (BIO-RAD, USA) were mixed with saliva samples and negative controls diluted 1:10 in PBS + 1% BSA + 10% skim milk, as well as the standards [(1:500 dilution factor IgG, 1:100 dilution factor IgM and IgA, all of them diluted in phosphate-buffered saline (PBS) + 1% bovine serum albumin (BSA)] and incubated for 1 hour. Wildtype-RBD-bound antibodies were detected using phycoerythrin (PE)-coupled goat anti-human IgG, IgA, and IgM antibodies (2 µg/mL, 204,009, 205,009, and 202,009, all from BIO-RAD) diluted in PBS + 1% BSA and incubated for 15 min. Finally, samples were analyzed using the Luminex 200 platform (R&D Systems, USA). All incubations were done at room temperature, on an orbital shaker, and protected from light. The plates were washed three times with PBS + 1% BSA between incubations. The final volume/well in incubations was 50 µL and, in the analysis, was 150 µL. The cutoff values were calculated using a receiver operating characteristic (ROC) curve where the specificity was prioritized. The threshold for assay positivity was defined using a ROC curve, prioritizing the specificity and set to 132 Fluorescence Intensity (FI).

### NAb measurement

As a proxy for measuring virus-NAbs, we used an in-house produced ELISA-based pseudo-neutralizing assay. This assay measures the interaction between the ACE-2 host receptor and Wildtype-RBD to estimate the degree of inhibition of virus-NAbs against Wildtype-RBD, as described previously (34). The threshold for virus-NAbs assay positivity was defined as 25% for NAb capacity and 420 international units (IU)/mL for NAbs levels using a ROC curve where the specificity was prioritized. To evaluate whether Omicron-infected individuals developed lower NAbs, we performed a variant-specific neutralization assay using BA.1 and BA.2 RBDs on randomly selected samples matched by sex, age, and time from infection or time from vaccination, accordingly. A total of 12 ng/mL and 18 ng/mL of BA.1 and BA.2 RBD were used. Of note, the correlation between the gold standard plaque reduction neutralization assay and the pseudo-neutralization assay has previously been found to be *r* = 0.9231 (34).

### IFN-γ release from T-cells and quantification

Cellular immune responses can easily be determined with an interferon-gamma (IFN-γ) release assay (IGRA), an *in vitro* blood diagnostics used to measure IFN-γ released by antigen-specific T-cells after stimulation with pathogen peptides. IFN-γ release is a reliable method of quantifying T-cell response after SARS-CoV-2 infection or vaccination (26, 35).

The SARS-CoV-2 IGRA stimulation tube set (ET 2606–3003, EUROIMMUN) was used according to the manufacturer’s instructions to stimulate T-cells against Wildtype-S1 protein peptides as previously described. Briefly, lithium heparin plasma was collected and stimulated overnight. Then, samples were aliquoted and stored at −80°C until IFN-γ determination. IFN-γ ELISA kit (ET 6841–9601, EUROIMMUN) was used to quantitatively determine IFN-γ levels released after stimulating T-cells against Wildtype-S1 protein following the manufacturer’s instructions as previously described (29). The threshold for assay positivity was set to 200 mIU/mL, according to the manufacturer.

### Statistics

Differences between groups in categorical variables were assessed using χ^2^ test. Statistical differences between groups from a categorical variable and immunological parameters were analyzed using Kruskal–Wallis test and multiple comparisons with Dunn’s correction. Statistical analyses between matched groups were performed using the Friedman test. IFN-γ correlation with IgG levels, NAbs, and days since the infection (positive RT-PCR) was analyzed using the Spearman’s rank correlation test. Analyses were stratified according to groups.

Modeling of serum IgG dynamics and NAbs dynamics was performed using generalized linear mixed models with two natural splines from the time from positive RT-PCR (in convalescent individuals). Modeling of saliva IgG and IFN- γ levels dynamics was performed using linear mixed models from the time from positive RT-PCR (in convalescent individuals). The interaction analyzed was between days from the time from positive RT-PCR (in convalescent individuals) and the study groups (convalescent cohort: group 2—unvaccinated SARS-CoV-2 convalescent individuals, group 5—vaccinated SARS-CoV-2 convalescent individuals (second dose), and group 6—vaccinated SARS-CoV-2 convalescent individuals (third dose). For all analyses, serum IgG, saliva IgG, NAbs, and IFN-γ levels were log10 transformed. *P*-values reported from mixed models were calculated using Type II Wald χ^2^ test.

The modeling was performed using R (version 4.1.0 for Windows, R Foundation for Statistical, Computing). The rest of the statistical analyses were performed using IBM SPSS Statistics for Windows v25 (IBM Corp., Armonk, NY) and GraphPad Prism version 9.0.0 (GraphPad Software, CA, USA). Significance levels are as follows: *P < 0.05, **P < 0.01, ***P < 0.001, ****P < 0.0001; *P* < 0.05 was considered statistically significant. All statistical test performed were two-sided.

## Data Availability

The data that support the findings of this study are available from the corresponding author upon reasonable request.

## SUPPLEMENTAL MATERIAL

Tables S1 and S2; Fig. S1 to S4 spectrum.00865-23-s0001.pdf Supplemental tables and figures.

ASM does not own the copyrights to Supplemental Material that may be linked to, or accessed through, an article. The authors have granted ASM a non-exclusive, world-wide license to publish the Supplemental Material files. Please contact the corresponding author directly for reuse.
