# Supplementary material for: Diverging humoral and cellular immune responses due to Omicron—a national study from the Faroe Islands
Source: Microbiol Spectr. 2023 Nov 1;11(6):e00865-23. doi: 10.1128/spectrum.00865-23 (PMC10714973; doi:10.1128/spectrum.00865-23)
Supplement: Tables S1 and S2; Fig. S1 to S4 — Supplemental tables and figures. [file spectrum.00865-23-s0001.pdf]

Supplementary Tables

Supplementary table 1. Levels of antibodies and IFN-γ release, stratified according to study group

**Serum**		Group 1 Unvaccinated naïve	Group 2 Unvaccinated convalescent	Group 3 Vaccinated naïve (second dose)	Group 4 Vaccinated naïve (third dose)	Group 5 Vaccinated convalescent (second dose)	Group 6 Vaccinated convalescent (third dose)
	Participants	N=88	N=82	N=11	N=103	N=102	N=107
**IgG, AU/ml**	Mean (SD)	120 (507)	1108 (2932)	8537 (10694)	30082 (30720)	49308 (46363)	87359 (62111)
	Median (range)	1 (1-4485)	170 (1-19277)	3163 (579-31548)	18205 ((633-179990)	30116 (2598-235618)	70149 (8721-320564)
**IgM, AU/ml**	Mean (SD)	12 (28)	12 (65)	1	25 (160)	23 (68)	27 (77)
	Median (range)	1 (0.3-128)	1 (1-580)	1	1 (1-1592)	1 (1-525)	1 (1-566)
**IgA, AU/ml**	Mean (SD)	6 (51)	34 (120)	124 (254)	285 (691)	1501 (1989)	1406 (1445)
	Median (range)	1 (1-477)	1 (1-709)	1 (1-755)	1 (1-4158)	846 (1-10441)	912 (1-8983)
**Neutralizing antibodies, IU/ml**	Mean (SD)	148 (464)	4376 (17722)	46962 (79170)	153368 (3910)	206723 (268533)	383022 (505104)
	Median (range)	1 (1-3943)	165 1-133650	7955 (1325-249960)	83189 (1380-1103470)	112021 (1852-1910348)	234532 (1-2000000)
**IFN-γ release from T-cells, mIU/ml**	Mean (SD)	327 (2117)	1904 (4244)	3862 (7067)	2444 (3209)	5743 (9918)	4446 (4650)
	Median (range)	1 (1-19628)	931 (123-35753)	4866 (54-24270)	1424 (29-18660)	3445 (336-65352)	2871 (1-23959)
**Saliva IgG, FI**	Mean (SD)	114 (465)	214 (739)	1288 (1842)	4253 (3910)	4862 (4339)	8438 (6618)
	Median (range)	39 (29-3819)	59 (32-6069)	689 (115-6236)	2724 (129-21871)	3778 (90-19436)	7199 (100-25787)

The estimated threshold between antibody positive and negative samples was 225 AU/ml for serum IgG, 100 AU/ml for serum IgA, 200 AU/ml for serum IgM, 420 IU/ml for neutralizing antibodies, 200 mIU/ml for IFN-γ release, and 200 FI for saliva IgG

Supplementary table 2. Characteristics of the Wildtype-infected individuals, sampled from July 2020 to January 2021

	Group W<br>Unvaccinated convalescent (Wildtype variant)*	
**Total (n)**	*n=235*	
**Sex, n (%)**	n=235	
Female	125	53.2
Male	110	46.8
**Age** (years), median (IQR)	33.9	24.0-50.1
**BMI (kg/m^2),** median (IQR)	n=198	
	25.0	22.3-28.0
**Smoking, n(%)**	*n=205*	
Ever	86	42
Never	119	58
**Daily medication use, n (%)**	*n=195*	
Yes	50	25.6
No	145	74.4
**Self-reported chronic disease[a], n (%)**	*n=199*	
Yes	68	34.2
No	131	65.8
**Days from RT-PCR test/Wildtype infection to blood sample,** median (IQR)**	28	24.0-36.0

[a]Asthma, heart disease, carnitine transporter deficiency, inflammatory bowel disease, hypertension, hypercholesterolemia, chronic obstructive pulmonary disease, type 2 diabetes; *all convalescent individuals were infected between 29 Jul 2020 to 08 January 2021.

Supplementary Figures

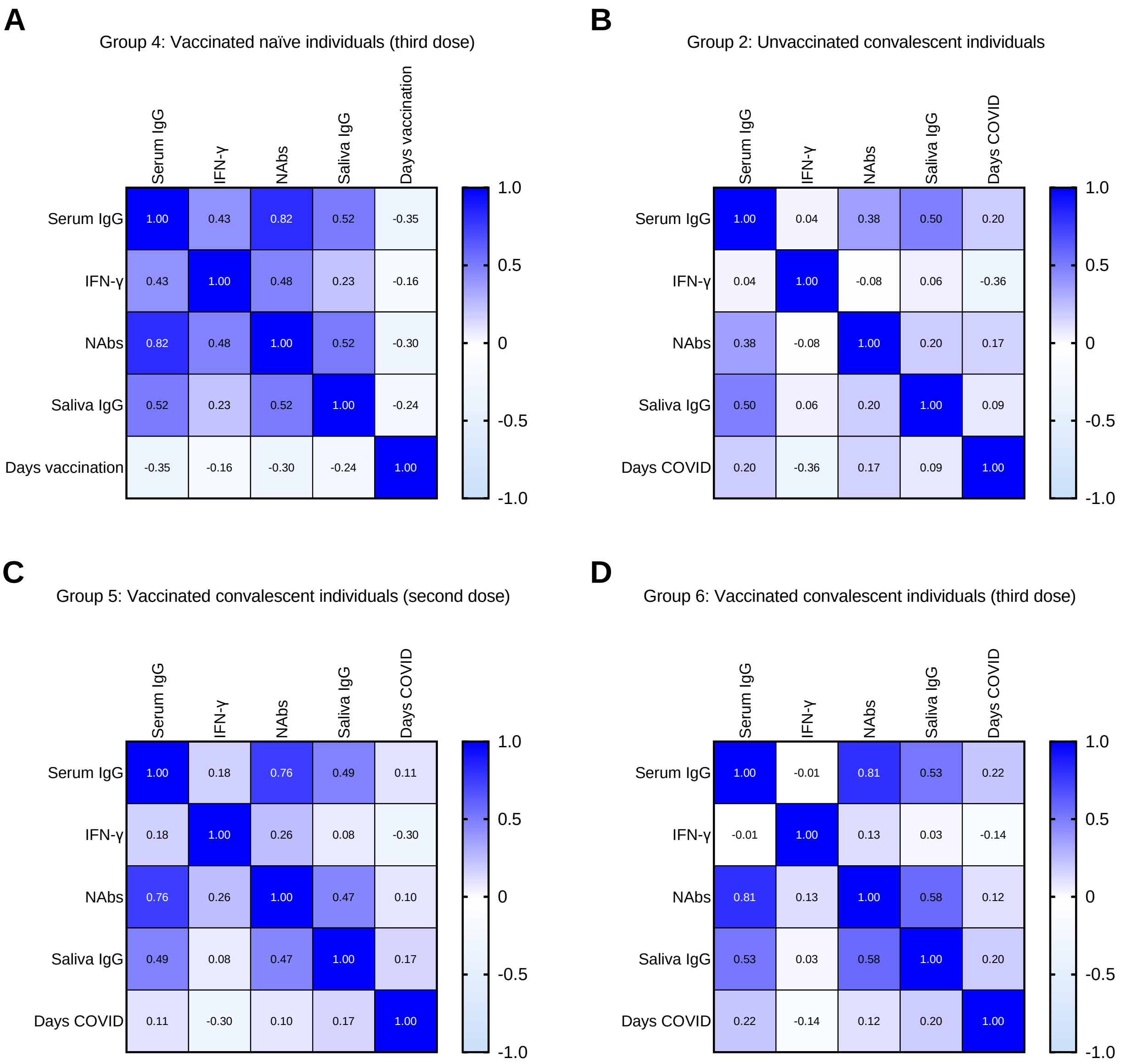

Supplementary Figure 1. Correlation between serum IgG, IFN-γ release, neutralizing antibodies (NAbs), saliva IgG and days since vaccination (group 4) or days since COVID infection (groups 2, 5, and 6). The values represented are Spearman's correlation coefficients. Spearman's correlation coefficients above 0.21 showed a significant correlation ($p < 0.05$). The participants are divided into four groups based on SARS-CoV-2 infection and/or vaccination: Group 4: vaccinated naïve individuals (third dose) (n=103); Group 2: unvaccinated convalescent individuals (n=82); Group 5: vaccinated convalescent individuals (second dose) (n=102); and Group 6: vaccinated convalescent individuals (third dose) (n=107).

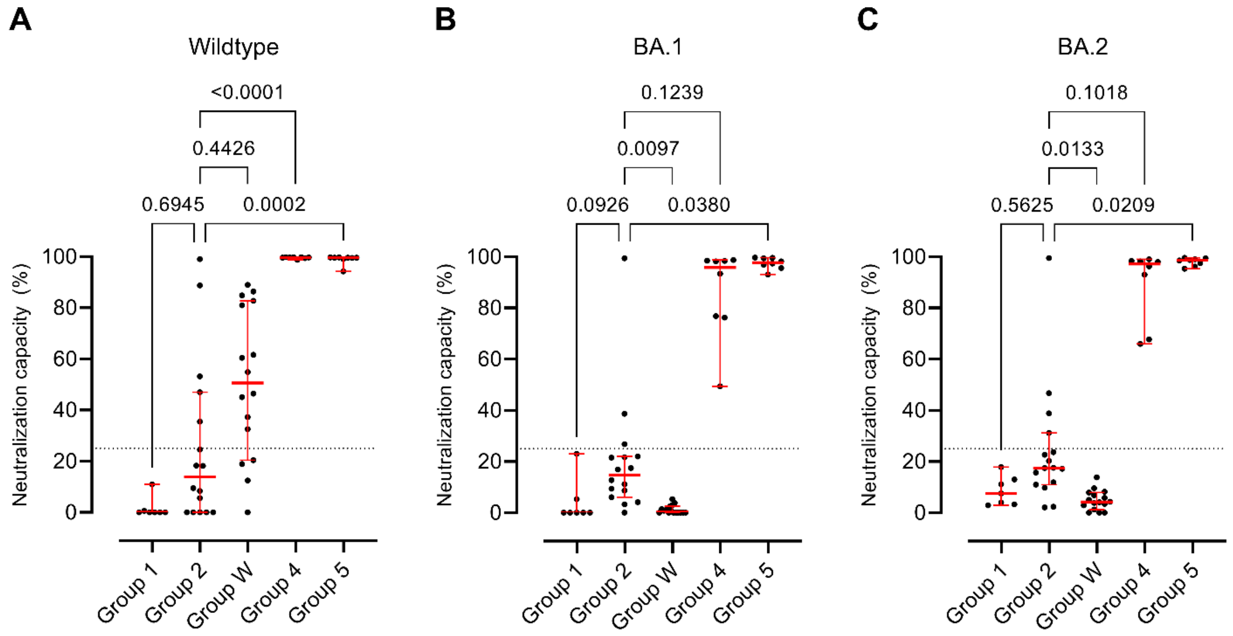

Supplementary Figure 2. Neutralization capacity against different RBD variants. Scatter plots display the median values and 95% confidence interval (CI) (red line and bar) of antibody neutralization capacity (%) against Wildtype RBD (A), Omicron BA.1 RBD (B), and Omicron BA.2 RBD (C). The participants are divided into five groups based on SARS-CoV-2 infection and/or vaccination: 1) unvaccinated naïve individuals (n=7); 2) unvaccinated Omicron-convalescent individuals (n=16); W) unvaccinated Wildtype-convalescent individuals (n=16); 4) vaccinated naïve individuals (third dose) (n=8); 5) vaccinated Omicron-convalescent individuals (third dose) (n=8). $p < 0.05$ was considered statistically significant by Kruskal–Wallis test followed by multiple comparisons with Dunn's correction compared to Group 2. The horizontal line represents the threshold for assay positivity (25%).

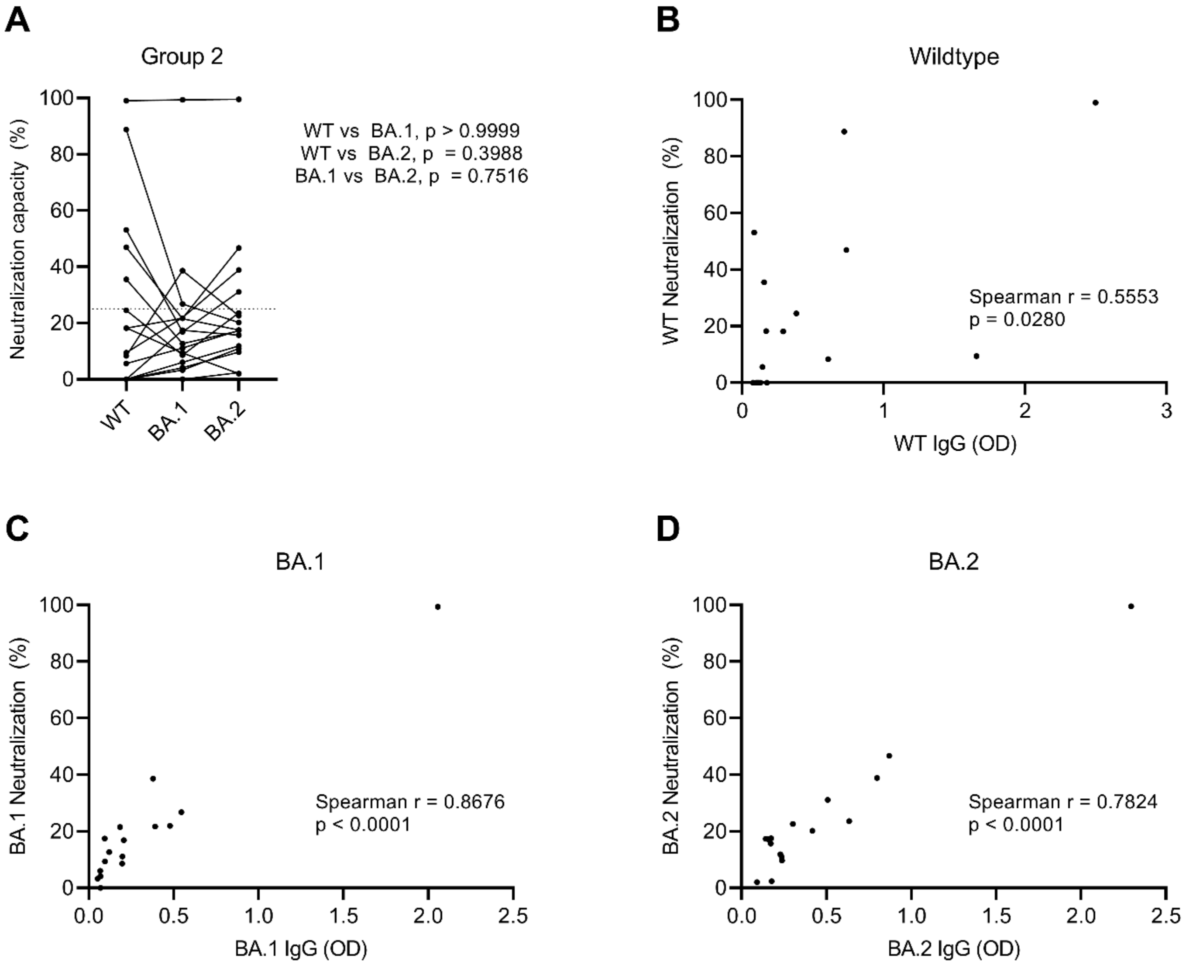

Supplementary Figure 3. Comparison between different RBD variants and between neutralization capacity and IgG antibodies. (A) Antibody neutralization capacity (%) of unvaccinated Omicron-convalescent individuals (n=16) against Wildtype RBD, Omicron-BA.1, and Omicron-BA.2 RBD. $p < 0.05$ was considered statistically significant by Friedman test followed by multiple comparisons with Dunn's correction. The horizontal line represents the threshold for assay positivity (25%). (B-D) Spearman correlation between antibody neutralization capacity (%) and IgG levels (Optical density, OD) of unvaccinated Omicron-convalescent individuals (n=16) against Wildtype RBD, Omicron BA.1, and Omicron BA.2 RBD $p < 0.05$ was considered statistically significant.

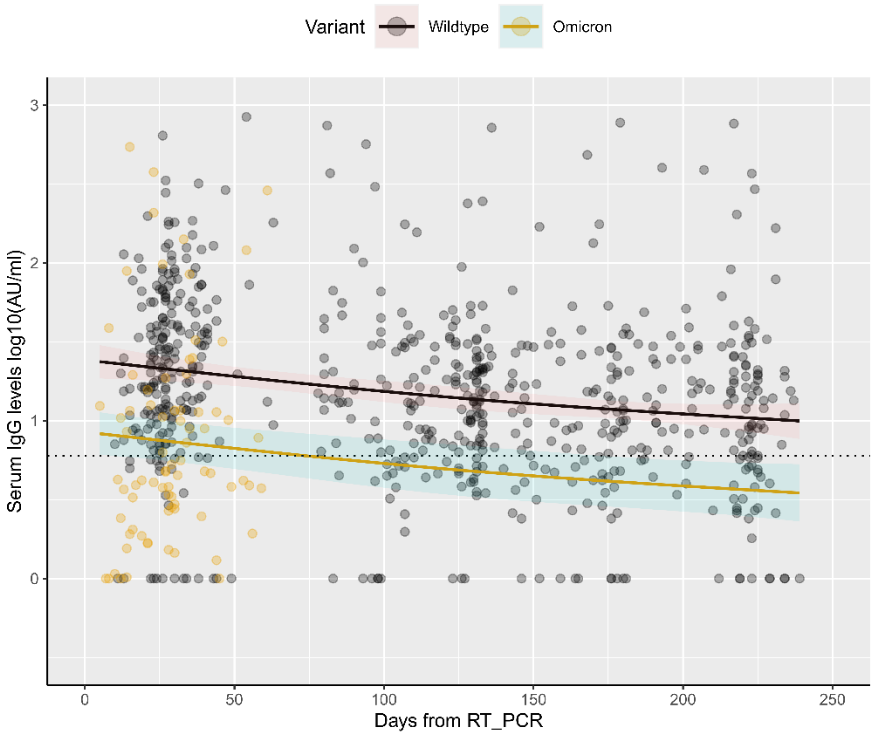

Supplementary Figure 4. Serum IgG levels dynamics over time in convalescent individuals using generalized linear mixed models. Distribution of serum IgG levels, represented in log10(AU/ml) over time (days from positive RT-PCR result). Circles represent observed levels for serum IgG. Solid lines represent predicted levels for serum IgG levels. Black and yellow colors represent unvaccinated Omicron-convalescent individuals (Group 2) and unvaccinated Wildtype-convalescent individuals (Group W), respectively. The horizontal dotted line represents the assay positivity threshold. Confidence interval (95%) is represented by the shadowed areas. Centre for the confidence interval is the predicted (mean) values.
